# Darier Ferrand dermatofibrosarcoma of the scalp with frontal intracranial extension

**DOI:** 10.11604/pamj.2014.18.264.4023

**Published:** 2014-07-30

**Authors:** Ali Akhaddar, Mohammed Lakouichmi

**Affiliations:** 1 Department of Neurosurgery, Avicenne Military Hospital, Marrakech, Morocco; 2 University of Mohammed V Souissi, Rabat, Morocco; 3 Department of Maxillofacial Surgery, Avicenne Military Hospital, Marrakech, Morocco

**Keywords:** Darier Ferrand, dermatofibrosarcoma, scalp, tumour

## Image in medicine

A 53-year-old man, previously healthy, presented with a slow-progressive enlarging mass in the midfrontal area which had developed 14 years previously without headache. On physical examination, this nodular reddish mass (11 x 7 x 5 cm) was fluctuant to pressure and tethered to the underlying frontal bone (A). There was no neurological deficit and no superficial lymph nodes. Cranial computed tomography scan showed an irregular median calvarial defect with poorly bony defined margins and left frontal sinus invasion (B-C). Magnetic resonance imaging revealed intracranial extension of the tumour which invaded the dura and the superior longitudinal sinus (D-E). Local biopsy was performed and histological study consisted with a dermatofibrosarcoma. In this specific case, radiotherapy was planned before surgical excision and craniofacial reconstruction. Dermatofibrosarcoma is a slow-growing mesenchymatous tumour of the skin with high local malignancy and great opportunity of recurrence. It usually occurs in the trunk or extremities. In the majority of cases the tumour remains asymptomatic for a long time. Head (scalp) and maxillofacial involvement is rare particularly with skull vault and intracranial extension. To improve local control after surgery, wide excision is recommended but difficult in significant anatomic region as in our case.

**Figure 1:**
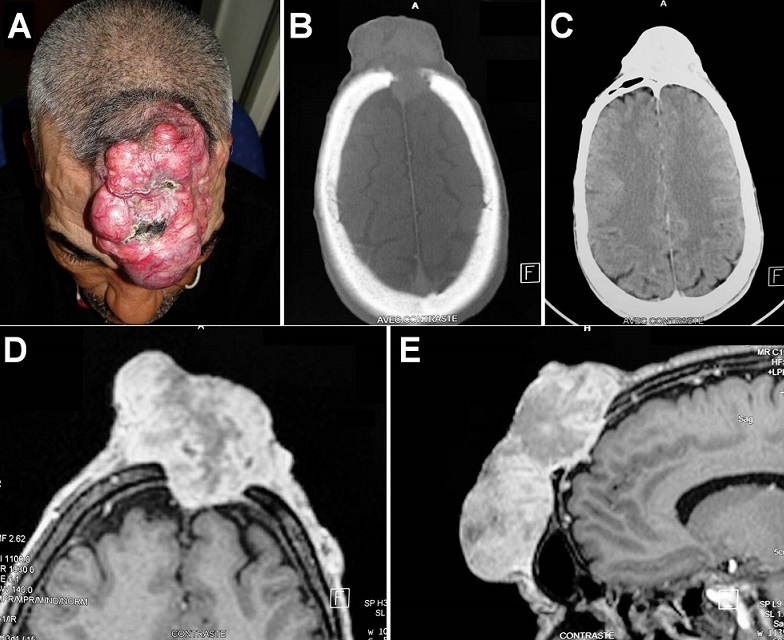
Patient photograph showing the midfrontal mass of the scalp (A). Axial cranial CT-scan revealing the frontal calvarial defect with poorly bony defined margins (B-C). Axial and sagittal T1-weighted MRI after gadolinium injection showing the enhanced soft heterogeneous extracranial process which invaded the dura and the superior longitudinal sinus (D-E)

